# A Local Proinflammatory Signalling Loop Facilitates Adverse Age-Associated Arterial Remodeling

**DOI:** 10.1371/journal.pone.0016653

**Published:** 2011-02-08

**Authors:** Mingyi Wang, Gaia Spinetti, Robert E. Monticone, Jing Zhang, James Wu, Liqun Jiang, Benjamin Khazan, Richard Telljohann, Edward G. Lakatta

**Affiliations:** 1 Laboratory of Cardiovascular Science, National Institute on Aging, National Institutes of Health, Baltimore, Maryland, United States of America; 2 Casa di Cura, MultiMedica-IRCCS, Milano, Italy; Istituto Dermopatico dell'Immacolata, Italy

## Abstract

**Background:**

The coincidence of vascular smooth muscle cells (VSMC) infiltration and collagen deposition within a diffusely thickened intima is a salient feature of central arterial wall inflammation that accompanies advancing age. However, the molecular mechanisms involved remain undefined.

**Methodology/Principal Findings:**

Immunostaining and immunoblotting of rat aortae demonstrate that a triad of proinflammatory molecules, MCP-1, TGF-β1, and MMP-2 increases within the aortic wall with aging. Exposure of VSMC isolated from 8-mo-old rats (young) to MCP-1 effects, via CCR-2 signaling, both an increase in TGF-β1 activity, up to levels of untreated VSMC from 30-mo-old (old) rats, and a concurrent increase in MMP-2 activation. Furthermore, exposure of young VSMC to TGF-β1 increases levels of MCP-1, and MMP-2 activation, to levels of untreated VSMC from old rats. This autocatalytic signaling loop that enhances collagen production and invasiveness of VSMC is effectively suppressed by si-MCP-1, a CCR2 antagonist, or MMP-2 inhibition.

**Conclusions/Significance:**

Threshold levels of MCP-1, MMP-2, or TGF-β1 activity trigger a feed-forward signaling mechanism that is implicated in the initiation and progression of adverse age-associated arterial wall remodeling. Intervention that suppressed this signaling loop may potentially retard age-associated adverse arterial remodeling.

## Introduction

Coincident collagen deposition and vascular smooth muscle cell (VSMC) cellularity within a diffusely thickened arterial intima is a salient feature of species-wide mammalian arterial wall remodeling that accompanies aging [1]–[3]. These age-associated alterations create a metabolically active microenvironment, especially in the thickened intima, and render it fragile, and vulnerable to pathologic stimuli, e.g., a high cholesterol diet, in older persons [1]–[7].

Novel insights into the cellular and molecular basis of this risk-prone niche have been recently reported. With advancing age, the proinflammatory biomolecules: monocyte chemoattractant protein-1 (MCP-1), matrix metalloproteinase type-II (MMP-2), and its activator calpain-1, and transforming growth factor-beta 1 (TGF-β1) become elevated within the arterial wall in numerous species, including humans [8]–[14]. Moreover, MCP-1, MMP-2, and TGF-β1 are also markedly elevated in early passage VSMC isolated from old vs. young aortae [11], [13]. Exposure of early passage isolated VSMC from young rat aortae to MCP-1 or MMP-2 shifts their phenotype to one resembling old untreated cells [11], [13]. Specifically, MCP-1 increases the invasive capacity of young VSMC to the level of their old counterparts; exposure of VSMC to MMP-2 enhances the levels of activated TGF-β1, and consequently increases the production of collagen types I and III, up to that of old cells [11], [13]. These findings suggest that an MCP-1/MMP-2/TGF-β1 signaling loop may be operative and a key to the collagen production and intimal invasion by VSMC, contributing to the concurrence of fibrosis and cellularity in the thickened intima of the aged arterial wall. Mutual interactions of these molecules both in vitro and in vivo form a putative signaling loop that is implicated in the histopathological features of the aged arterial wall.

In the present study, we demonstrate, for the first time, that: 1) co-expression of MCP-1 and TGF-β1 is increased within the aged aortic wall; 2) MCP-1 treatment increases activation of TGF-β1 and its downstream signaling molecules, to produce collagen; 3) MCP-1 also increases the invasive capability of young VSMC in an MMP-2-dependent manner, to that level of old untreated cells; 4) following TGF-β1 treatment of young cells, MCP-1 production, MMP-2 activation and VSMC invasion increase, reaching the levels of untreated old cells. We demonstrate the existence of this autocatalytic signaling loop that enhances collagen production and invasiveness of VSMC, and that it can be effectively disabled by si-MCP-1, a CCR2 antagonist, or MMP-2 inhibition. Thus, interventions aimed at any one of these local signaling loop elements may potentially retard adverse age-associated arterial remodeling.

## Results

### Increased MCP-1 and TGF-β1 co-localize within the aortic wall of old rats

**Figure 1:**
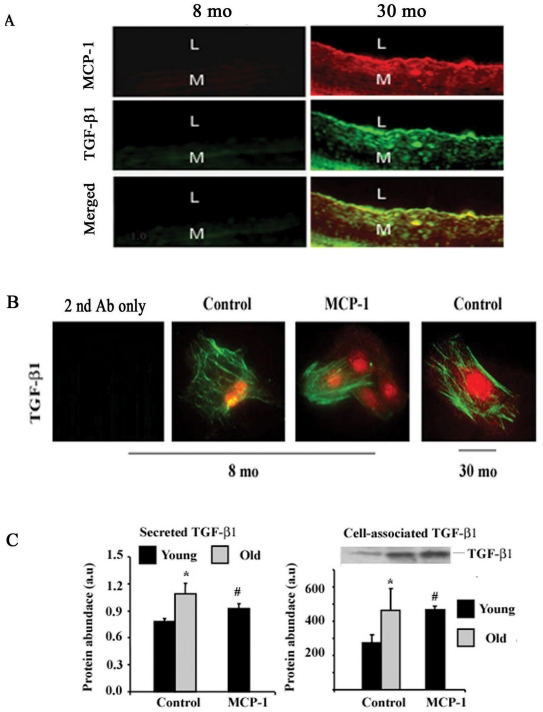
Age and MCP-1 effect on expression of TGF-β1. **A.** Immunofluorescence staining in rat aortic walls. MCP-1 (red), TGF-β1 (green), merged (yellow). Original magnification: X400. **B.** TGF-β1 (green) and α**-**SMA (red) immunocytostaining (n = 3, X400). **C.** ELISA for activated TGF-β1 protein (left panel) and Western blots (right panel, inset) and average data (right panel). Early passage young and old VSMC were treated with or without MCP-1 (50 ng/ml) for 24 hours. *p<0.05, old vs. young control; #p<0.05, young with treatment of MCP-1 v.s. young control.

FXBN rats are a well-characterized animal model of cardiovascular aging with similarities to the human anatomic and physiologic phenotype. MCP-1 and TGF-β1 increase within the aortic wall, and both co-immunolabel with alpha-smooth muscle actin (α-SMA), a marker of VSMC [9], [11]. Double immunolabelling shows that aortic MCP-1 (red), TGF-β1 (green) immunolabeling, and their co-localization (merged image: yellow color) increase in the aged rat aortic wall, predominantly within the thickened intima in rats (**Figure 1A** **, bottom right panel**).

### MCP-1 induces VSMC TGF-β1 protein expression, activation, and secretion

To further investigate a link between MCP-1 and TGF-β1, we isolated VSMC from young and old rats. Prior studies demonstrated that cultured early passage (p3**–**5) VSMC maintain an age-related typical inflammatory profile in vivo such as elevated levels of MMP-2, MCP-1 and calpain-1[11], [13], [14]. **Figure S1** shows further that early passage (p3) VSMC also retain typical age-associated characteristics as in vivo cells, including cytoskeleton proteins, a-SMA, vimentin, and desmin. MCP-1 treatment of early passage cells (p3) from young rats for 24 hr enhances levels of TGF-β1 protein signal (**Figure 1B**), up to that of old untreated cells. Treatment with MCP-1 induces cell-associated TGF-β1 protein expression in young rats, to an extent equivalent to that of old, untreated cells (**Figure 1C, right panel**). Furthermore, TGF-β1 secretion, as assessed via an ELISA, is significantly higher in untreated old vs. young VSMC (**Figure 1C, left panel**). MCP-1 also induces TGF-β1 protein secretion from young aortic VSMC, up to levels of untreated old cells (**Figure 1C, left panel**).

**Figure 2:**
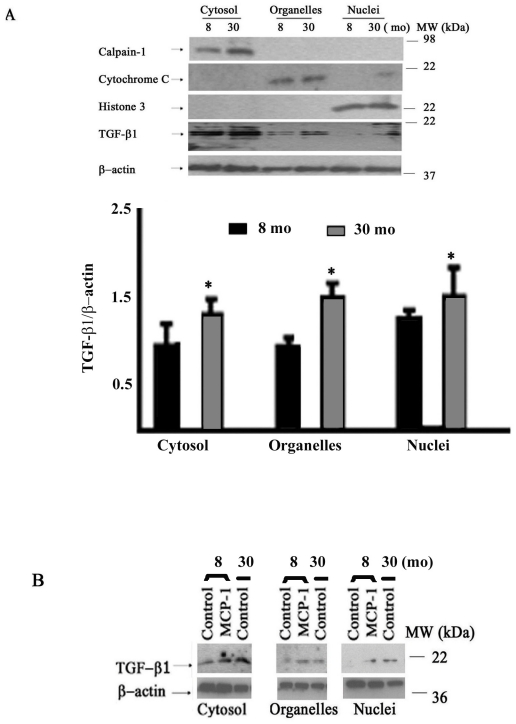
Age effects on subcellular distribution of TGF-β1. **A.** Western blots of TGF-β1 subcellular fractions with aging (upper panels) and average data of subcellular TGF-⇓1 protein fractions lower panel). **B.** Representative Western blots of TGF-β1 subcellular fractions with aging and MCP-1 treatment. Similar results were obtained from at least 3 separate experiments. Early passage young and old VSMC were treated with or without MCP-1 (50 ng/ml) for 24 hours. *p<0.05, old vs. young.

To define the location of cellular TGF-β1 within VSMC, we performed Western blotting of the cellular protein subfractions including cytosol, organelles, and nuclei of VSMC. TGF-β1 protein significantly increases in all subcellular fractions in old versus young cells (**Figure 2A**). Interestingly, following MCP-1 treatment, subcellular activated TGF-β1 increased in young VSMC in nuclei, cytosol, and organelle compartments, up to the level of old untreated cells (**Figure 2B**).

### MCP-1 also modulates the CCR2/MMP-2/TGF-β1 signaling axis

**Figure 3:**
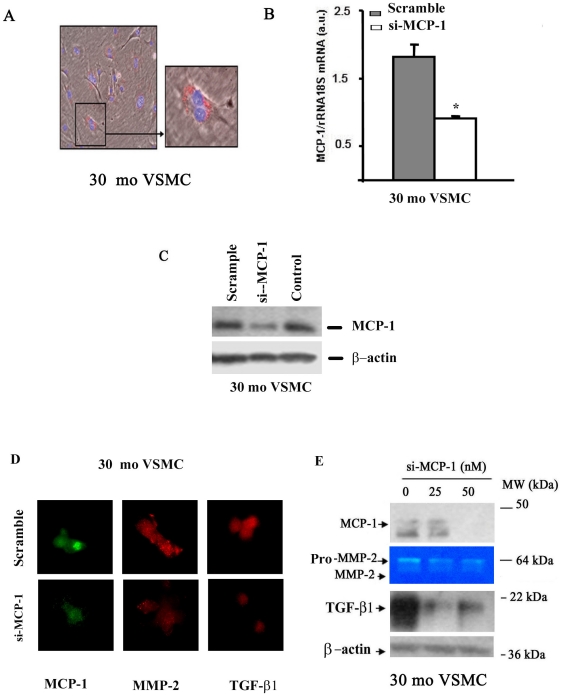
siRNA MCP-1. **A.** Photomicrograph of MCP-1 silenced VSMC. Inset showing enlarged image (X1000). **B.** qRT-PCR. *p<0.05, siRNA MCP-1 vs. scramble in 30 mo VSMC. **C.** Western blots of MCP-1 in 30 mo VSMC (n = 3). **D**. Immunofluoscence staining of MCP-1 (green), MMP-2 (red), and TGF-β1 (red) (X400) in 30 mo VSMC treated with scramble (upper panels) and MCP-1 (50 nm, lower panels) siRNA. **E.** Representative of Western blots for MCP-1 and TGF-β1 and PAGE gelatin zymograph. Similar results were obtained from at least 3 separate experiments. *p<0.05 vs. scramble control.

Our prior studies have demonstrated that MMP-2 and MCP-1 concurrently increase within the aged rat arterial wall, and that MMP-2 activates TGF-β1 signaling via proteolysis [11], [13]. An age-associated increase in MMP-2 levels and activity may be linked to increases in both MCP-1 and TGF-β1 and to increased activation of the latter. We employed siRNA of MCP-1 to determine its effects on MMP-2 activity within old VSMC. The microphotograph in **Figure 3A** indicates the transfection efficiency and location (perinuclear space, enlarged image). RT-PCR shows that si-MCP-1 substantially decreases MCP-1 mRNA abundance (**Figure 3B**). Correspondingly, Western blot analysis indicated that si-MCP-1 substantially decreases MCP-1 protein abundance (**Figure 3C**). Importantly, after silencing, not only is MCP-1 protein reduced, but MMP-2 and TGF-β1 protein and activation is also simultaneously reduced in a dose-dependent fashion (**Figure 3D & E**).

**Figure 4:**
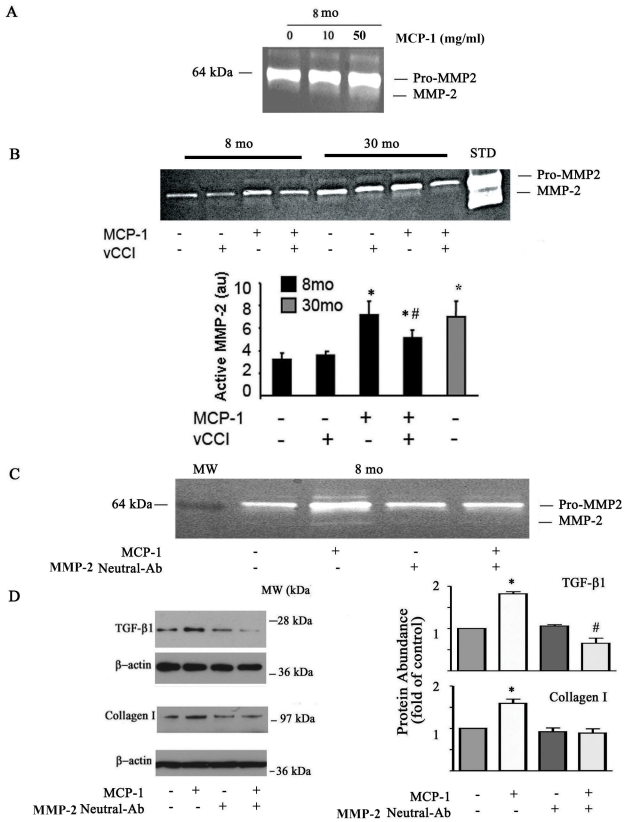
Profile of MMP-2, TGF-β1 and downstream signaling molecules with aging and MCP-1 intervention. **A**. Gelatin zymograph. Early passage young VSMC were treated with or without MCP-1 for 24 hours. **B.** Gelatin zymograph (upper panel); and average data (lower panel). Early passage young and old VSMC were treated with or without MCP-1 (50 ng/ml) plus vCCI (100 ng/ml) for 24 hours. **C.** Gelatin zymograph (n = 3). Early passage young VSMC were treated with or without MCP-1 (50 ng/ml) plus neutralizing antibody against MMP-2 (2 µg/ml) for 24 hours. **D**. Representative Western blots of MCP-1 and collagen I after MCP-1 treatment (left panels) and average data (right panels). Early passage young VSMC were treated with or without MCP-1 (50 ng/ml) plus neutralizing antibody against MMP-2 (2 µg/ml) for 24 hours. *p<0.05, vs. young; #p<0.05, vs MCP-1 treatment alone.

**Figure 4A** shows that MCP-1 treatment of VSMC increases MMP-2 activation (lowest weak bands) in a dose-dependent manner. Further, the MCP-1-treatment associated MMP-2 activation in VSMC is substantially reduced by vCCI, a blocker of the cognate MCP-1 receptor (**Figure 4B**).

MMP-2 activity links MCP-1 to TGF-β1 signaling. PAGE zymogram indicates that MCP-1 in young VSMC markedly increases MMP-2 activation, which is abolished by a neutralizing antibody against MMP-2 (**Figure 4C**). Western analysis shows that the MCP-1 markedly increases the activated form of TGF-β1, and significantly increases the expression of its downstream signaling molecule, procollagen type I, which is diminished by a neutralizing antibody against MMP-2 in young VSMC (**Figure 4D**). Notably, the abundance of protein and activity of TGF-β1 in old VSMC also decreases after MCP-1 silencing (**Figure 3 D&E**).

### MMP-2 signaling links MCP-1 to VSMC invasion

**Figure 5:**
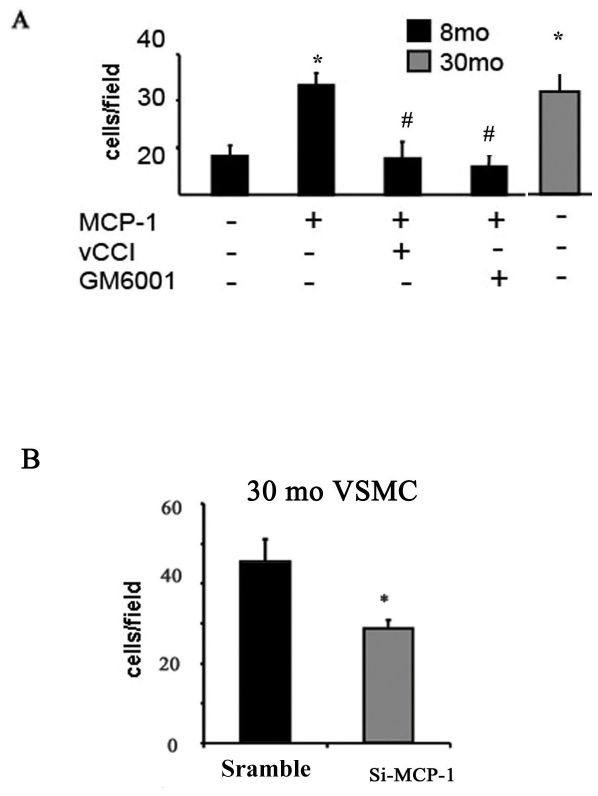
VSMC Invasion. **A.** VSMC treated with or without MCP-1 (50 ng/ml) plus vCCi (150 ng/ml) or GM 6001 (15 µM). *p<0.05, vs. young; #p<0.05, vs MCP-1 treatment alone. **B.** Old VSMC with or without si-MCP-1 (50 nM). *p<0.05 si-MCP-1 vs. scramble control.

Prior studies indicate that MCP-1/CCR-2 signaling and MMP-2 activation are both involved in the age-associated increase in the ability of rat VSMC to invade basement membranes [9], [11], [13]. Here, we performed an additional series of experiments to address the details of this interaction. **Figure 5A** shows that invasion through a matrigel membrane of early passage VSMC from aged rats in response to a PDGF-BB gradient is increased by over 60% compared to that of VSMC from young rats. MCP-1 treatment (50 ng/ml, 24 hour) enhances the ability of VSMC isolated from young rats to invade the matrigel membrane by 40%, i.e., to that level of untreated old VSMC (**Figure 5A**). This effect was blunted not only by the CCR2 blocker, vCCI (150 ng/ml), but importantly, also by the metalloproteinase inhibitor, GM 6001 (15 µM) (**Figure 5A**). Importantly, MCP-1 silencing substantially decreases the invasive capability of older cells (**Figure 5B** **).**

### TGF-β1 increases MCP-1 expression, MMP-2 activation, and VSMC Invasion

**Figure 6:**
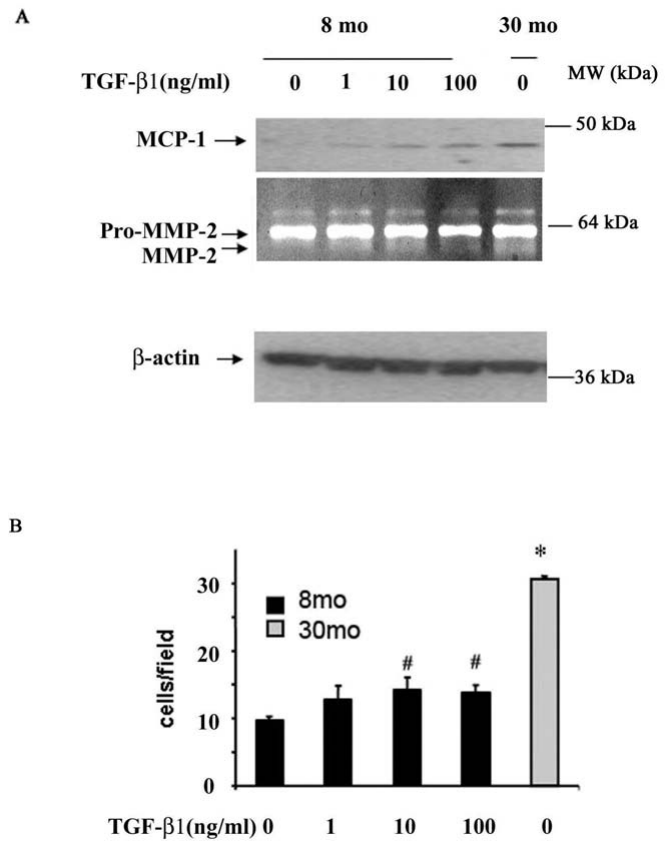
Effects of TGF-β1 on young VSMC. **A.** Representative Western blots of MCP-1 (upper panel) and β-actin (lower panel) and zymograph (middle panel). **B.** VSMC invasion. Early passage young and old VSMC were treated with or without TGF-β1 (1, 10, 100 ng/ml) for 24 hours. *p<0.05, vs. young; #p<0.05, TGF-β1 treatment vs young.

The results of the above experiments are attributable to an MCP/MMP-2/TGF-β1 signaling loop and suggest that the progressive arterial intimal thickening and accumulation of cellularity and fibrosis within the thickened intima during aging may involve this feed-forward signaling. If this were the case, activated TGF-β1 should lead to an increase in MCP-1 protein, MMP-2 activation, and affect the VSMC invasion phenotype. **Figure 6A** demonstrates that exposure of young VSMC to TGF-β1 indeed increases both MCP-1 abundance and secreted activated MMP-2 in a dose-dependent manner, reaching levels of untreated old cells; and that exposure of young VSMC to TGF-β1 also enhances their invasive capacity, which approaches that of old untreated cells **(** **Figure 6B** **)**. Interestingly, RT-PCR show that exposure of young VSMC to TGF-β1 (100 ng/ml) for 24 h didn't affect levels of MCP-1 mRNA compared to control (1.85±0.41 vs.1.88±0.21, p>0.05).

## Discussion

**Figure 7:**
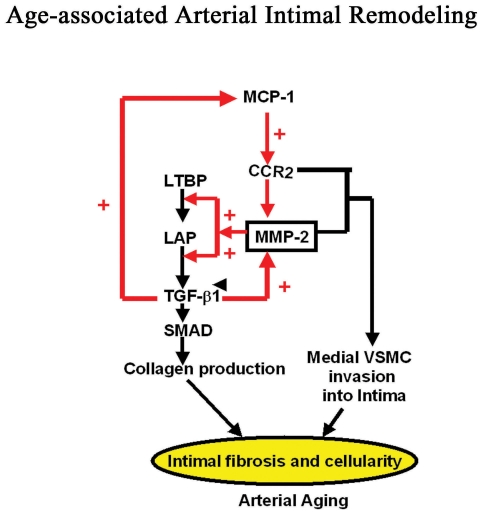
Simplified schematic diagram of a MCP-1/MMP-2/TGF-β1 loop signaling pathway.

The novel findings of this study support the idea that a feed-forward, MCP-1/MMP-2/TGF-β1, proinflammatory signalling loop triad, as depicted schematically in **Figure 7** **,** sustains processes that are associated with the arterial remodelling that accompanies advancing age.

Our results show that MCP-1 directly increases MMP-2 activation via its cognate receptor CCR-2. Active MMP-2 increases the active form of TGF-β1 by its proteolytic effects on its latent forms, LAP and LTBP-1 [13]. The active form of TGF-β1 enhances itself via a feed-forward effect, by activating both MCP-1 and MMP-2. Active TGF-β1 increases the activity of MMP-2, via a direct effect on its activation, and an indirect effect via MCP-1/CCR2 signaling. MCP-1 maintains its level indirectly via its effect to activate MMP-2, which activates TGF-β1, which, in turn, increases MCP-1. Thus, each of these molecules, MCP-1, MMP-2, and TGF-β1, are part of a feed-forward loop that sustains each others level or activity and drives intimal VSMC cellularity and fibrogenesis, salient features of arterial aging.

The triad of proinflammatory molecules not only are maintained in their levels of translation and activation in the aging arterial wall or cells, but also may be linked at the transcription level. Our prior studies show that the transcription levels of MCP-1, MMP-2, and TGF-β1, increases in the aged arterial wall *in situ* and in aged VSMC *in vitro* [9]-[13]. Most recently, a micro-RNA, i.e., MiR-21, has emerged as a potential modulator of transcriptional coregulation of TGF-β1 and MMP-2 [15], [16].

Fibrotic signaling via the MCP-1/MMP-2/TGF-β1 cascade is increased within both the old arterial wall and early passage VSMC isolated from the aortic wall, and may modulate progressive arterial fibrogenesis with aging. Indeed, exposure of young VSMC to exogenous MCP-1 enhances MMP-2 activation and consequently elevates TGF-β1 activation and collagen production up to levels of old untreated cells. This can be substantially reduced by a CCR-2 blocker or an MMP-2 inhibitor. Importantly, age-elevated endogenous TGF-β1 and its consequent signaling product collagen type I in old VSMC return to levels of young cells after silencing of MCP-1, blockade of CCR-2 signaling, or inhibition of MMP-2 activation. These findings further expand the prior concept that MMP-2 activation induces matrix production via TGF-β1 activation in VSMC with aging [13]. In addition, our findings, for the first time, suggest that TGF-β1, activated intracellularly and within the nucleus, is increased in VSMC with aging, as in hepatic stellate cells and hepatocytes after injury [13], [17]-[19], and may play a role in the initiation and progression of VSMC aging via epigenetic modulation in an intracrine manner.

Our results demonstrate that MCP-1/MMP-2/TGF-β1 loop signaling not only promotes VSMC fibrogenesis, but also facilitates chronic VSMC invasion of the sub-endothelial space, contributing to intimal thickening. Our findings show that MCP-1 increases invasiveness of VSMC during aging via a triad effect: by activation of MMP-2 and by its chemoatractant signaling via the MCP-1 receptor, CCR2, as well as by activation of TGF-β1. The present and prior studies [11] show that in response to PDGF-BB (1) the invasiveness of MCP-1 treated young VSMC increases up to the level of untreated old VSMC, an effect that is abolished by GM6001 or vCCi; and (2) the enhanced invasiveness of old VSMC is blunted by MCP-1 silencing, vCCi, or GM-6001. Thus, current and prior findings [20] suggest that MCP-1 and MMP-2 are direct targets and mediators of TGF-β1-induced VSMC invasion.

Accumulating evidence indicates that sustained, age-associated increases in transcription, translation and activation of the triad of proinflammatory modulators MCP-1, MMP-2, and TGF-β1 [8]–[13], is linked to the arterial wall Ang II signaling cascade in rodents, nonhuman primates and humans [2], [3], [9], [14]. Indeed, the invasive capacity of young medial VSMC induced by both Ang II and its downstream signaling molecule, milk fat globule-EGF-8 (MFG-E8) mediated by MCP-1 signaling are increased, approaching the levels of untreated old cells [8], [21]. This Ang II orchestration of triad signaling potentially governs the initiation and progression of age-associated arterial intimal cellularity and collagen deposition in a feed-forward mechanism by which threshold amounts of MCP-1, MMP-2, or TGF-β1 activates the loop triad, triggering a positive developmental cascade. This feed-forward positive signaling loop, however, does not function in isolation, but is tightly controlled by a complex signaling network, including its suppression by insulin like growth factor binding protein-7 (IGFBP-7) and tissue inhibitor of metalloproteinases-2 (TIMP-2) signaling that change with aging [13], [21]. It is likely that, over the long period of time over which aging occurs a chronic imbalance of the signaling loop herein and other suppressive signaling enables the induction of VSMC invasion and collagen deposition within the arterial wall. Similar Ang II-related activation of molecular and cellular events also occurs within the neointima or atheroscleroma formation during the early stages of arterial injury and atherosclerosis and hypertension, respectively [22]–[30]. Thus, arterial wall aging could be construed as a journey into a subclinical vascular disease state, and targeting this feed-forward system is a novel potential approach to sustain arterial health with advancing age.

## Materials and Methods

### Animals

Male Fisher 344 crossbred Brown Norway rats (FXBN), 8-month-old (8mo: n = 20) and 30-month-old (30mo: n = 20), were obtained from the National Institute on Aging Contract Colonies (Harlan Sprague Dawley, Inc, Indianapolis, IN). All of the animals were sacrificed immediately by an overdose of sodium pentobarbital, and the thoracic aortae were immediately removed, isolated, and processed as described previously [9], [11], [13], [14]. The animal protocols (EGL-Ra210) used were approved by the Institutional Animal Care and Use Committee of the Gerontology Research Center and complied with the guide for the care and use of laboratory animals (NIH publication No. 3040-2, revised 1999).

### Immunofluorescence staining

Thoracic aortae frozen sections and cultured VSMC were incubated overnight at 4°C with primary antibodies diluted in accordance with the manufacturer's instructions. Tissue sections were further incubated with secondary, fluorescence conjugated antibodies, and photomicrographs were acquired by a computer-imaging program (MetaMorph Imaging System, Universal Imaging Corp., PA). All immunostaing was performed on frozen aortic sections from at least 4 animals. The source and characteristics of primary antibodies used were listed in **Table 1**.

**Table 1:** Primary Antibodies.

Antibody	Specie	TiterBlotting	TiterStaining	Sources
MCP-1	M	1∶1000	1∶50	Chemicon Intern. Inc., CA
TGF-β1	R	1∶200	1∶50	Santa Cruz, CA
Calpain-1	R	1∶2500		Sigma, St. Louis, MO
Cytochrome	M	1∶1000		Cell Signaling Technology Inc. MA
Histone 3	M	1∶1000		Cell Signaling Technology Inc. MA
MMP-2	M		1∶50	Chemicon Intern. Inc., CA
Collagen I	R	1∶1000		Rockland Immunochemicals, Inc., PA
β-actin	M	1∶10000		Sigma-aldrich, Inc. MO

M = mouse; and R = rabbit.

### VSMC Isolation, culture, and treatment

Vascular smooth muscle cells were enzymatically isolated and cultured as previously described [11]. Briefly, F344XBN rat thoracic aortas were rinsed in Hanks balanced salt solution (HBSS) containing 50 µg/mL penicillin, 50 µg/mL streptomycin and 0.25 µg/mL amphotericin B (Gibco). After digestion for 30 min in 2 mg/mL collagenase I solution (Worthington Biomedical, Freehold, New Jersey) at 37°C, the adventitia and intima were removed from the vessel media layer, which was placed overnight in complete medium (DMEM plus 10% FCS). On day 2 the vascular media was further digested with 2 mg/mL collagenase II/0.5 mg/mL elastase (Sigma) for 1 hour at 37°C, and the isolated cells were washed and plated in complete medium. In all cases, >95% of cells stained positive for α-smooth muscle actin (α-SMA).

Early passage (p3) VSMC were treated with or without MCP-1(1, 10, 50 and 100 ng/ml, R&D research Inc., MN), TGF-β1 (1, 10, and 100 ng/ml, R&D research Inc., MN), vCCI (150 ng/ml, R&D research Inc., MN), and a neutralizing antibody against MMP-2 (2 µg/ml, R&D research Inc., MN) for 24 hours in 0.1% FBS for PAGE zymography and Western blot analysis.

### Western Blotting and Gel Zymography

VSMC plated on 100 mm petri dishes, were washed twice with cold PBS, 80 µL of lysis buffer (LB: 20 mmol/L Tris-HCl, pH 7.5; 150 mmol/L NaCl; 1 mmol/L Na_2_EDTA, 1 mmol/L EGTA; 1% Triton; 1 µg/mL leupeptin; 1 mmol/L PMSF) were added, and the cells scraped from the plate and transferred to 1.5 mL tubes. Sub-cellular protein fractions were isolated according to the manufacturer's instructions (ProteoExtract kit, EMD Biosciences, La Jolla, CA). Fifteen µg of proteins were resolved by SDS-PAGE and transferred onto PVDF membranes (Immobilon). HRP-conjugated IgG (Amersham Pharmacia Biotech) were used as secondary antibodies and detected with SuperSignal West Pico Chemiluminescent Substrate (Pierce Biotechnology). The source and characteristics of primary antibodies used were listed **in** **Table 1**.

### Real time PCR

RNA was extracted from VSMC using Trizol reagent (Invitrogen) following the manufacturer's instructions. RNA (500 ng) was reverse transcribed for 30 minutes at 48°C using random hexonucleotides according to the manufacturer's instructions (Applied Biosystems). Real-time PCR of MCP-1 was performed following the SYBRGreen PCR based protocol as previously described [11]. Briefly, each sample has been tested in quadruplicate. The reaction conditions were: 10 min at 95°C (one cycle), and 15 sec 95°C, 20 sec 60°C, and 30 sec 72°C (40 cycles). Gene-specific PCR products were continuously measured by means of an ABI PRISM, 7900 HT Sequence Detection System (PE Applied Biosystem) and the PCR product sizes were verified by agarose gel electrophoresis. Samples were normalized to the expression of the “housekeeping” gene, the rRNA 18s, which did not change with age. Data are expressed as the mean quantity calculated using the following formula: quantity = 10^–(Ct-Y intersept)/slope value)^, where Ct represents the threshold cycle value.

### siRNA

For siRNA protocols, pre-annealed purified siRNA probes were obtained from Dharmacon and were rehydrated prior to transfection using their standard protocol. The siRNA used were a pool of four specific RNAs targeting the MCP-1 sequence. A scramble siRNA pool of probes was used as control. siRNA probes (25 and 50 nM) were transfected using Gene Silencer (Gene Therapy Systems). After 24 hours of transfection, VSMC were harvested, RNA or protein extracted for further analysis, or cells were fixed and processed for immunofluorescence staining.

### Enzyme-linked immunosorbent assay (ELISA)

Secreted TGF-β1 activity from young and old VSMC treated with or without MCP-1 (50 ng/ml) for 24 hours was assayed with Quantikine ELISA kit (Cat#: D B100B; R & D Systems Inc. MN).

### VSMC invasion

Modified Boyden chambers were equipped with 8 µm pore-size polycarbonate filters (Costar) coated with Matrigel (BD Biosciences). VSMC were pre-treated with MCP-1 (50 ng/mL) for 24 hours in 2.5% FBS. MCP-1 (50 ng/ml) with and without vCCi (100 ng/ml, R&D System Inc. MN) and GM 6001 (15 µM, Calbiochem International, CA), PDGF-BB (10 ng/mL) or BSA (control) was added to the lower chamber in 220 µL of MM (MM: DMEM, 0.1% BSA). VSMC (10^6^/mL) were placed in the upper chamber in 200 µL of MM. The assay was stopped after 4 hours at 37°C, and cells that had invaded the membrane and migrated through the filter were fixed on the lower side of the filter, and stained using the HEMA3 system (Curtin Matheson Scientific). Four random fields were counted at 400X magnification for each chamber.

### Statistical analysis

All results are expressed as the mean ± SEM. Statistical analysis was performed via a T-test when two groups were analyzed, or via an ANOVA, followed by a Bonferroni post hoc test for multiple comparisons. A p value of <0.05 was taken as statistically significant.

## Supporting Information

Figure S1 **Cytoskeletal remodeling in the aortic wall and primary cultured VSMC with aging. A.** Representative Western blots of α-SMA (upper), vimentin (middle), and desmin (lower) within the arterial wall (left panels) and VSMC (middle and right panels). **B.** Average data of α-SMA (upper panel), vimentin (middle panel), and desmin (lower panel). *p<0.05, vs. 8 mo.
(TIF)
