# Supplementary figures and images for: A Local Proinflammatory Signalling Loop Facilitates Adverse Age-Associated Arterial Remodeling
Source: PLoS One. 2011 Feb 8;6(2):e16653. doi: 10.1371/journal.pone.0016653 (PMC3035650; doi:10.1371/journal.pone.0016653)

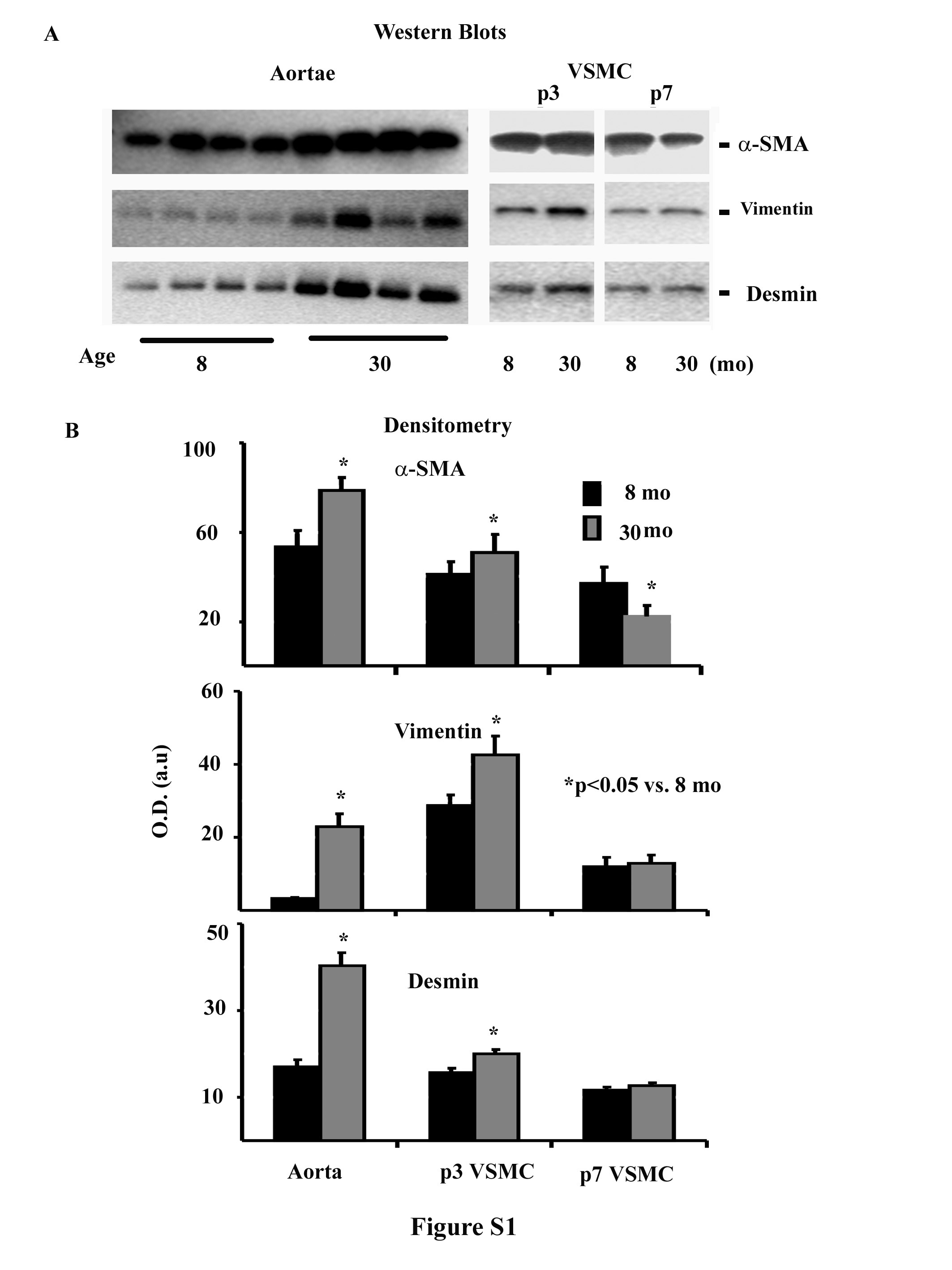

Supplement: Figure S1 — Cytoskeletal remodeling in the aortic wall and primary cultured VSMC with aging. A. Representative Western blots of α-SMA (upper), vimentin (middle), and desmin (lower) within the arterial wall (left panels) and VSMC (middle and right panels). B. Average data of α-SMA (upper panel), vimentin (middle panel), and desmin (lower panel). *p<0.05, vs. 8 mo. (TIF) [file pone.0016653.s001.tif]
